# Comparative Evaluation of Perceived Cognitive Load Across Two Academic Cycles in an Undergraduate Obstetrics and Gynecology Course With an Evolving Clinical Tutor Role: Comparative Student Feedback Analysis

**DOI:** 10.7759/cureus.104590

**Published:** 2026-03-03

**Authors:** Mariam Siam, Orjwan Albakri, Jeyaseelan Lakshmanan, William Atiomo, Mutairu Ezimokhai

**Affiliations:** 1 Medical Education and Simulation, Mohammad Bin Rashid University of Medical and Health Sciences, Dubai, ARE; 2 Biostatistics and Epidemiology, Mohammad Bin Rashid University of Medical and Health Sciences, Dubai, ARE; 3 Obstetrics and Gynaecology, Mohammad Bin Rashid University of Medical and Health Sciences, Dubai, ARE

**Keywords:** clinical tutors, cognitive load theory, curriculum evaluation, near-peer teaching, obstetrics and gynecology, self-perceived learning, undergraduate medical education

## Abstract

Background

Near-peer teaching in medical education is commonly described as informal, voluntary, or extracurricular, and most often involves senior medical students teaching junior peers. Existing literature demonstrates benefits such as improved learner comfort, engagement, and perceived approachability; however, these findings predominantly arise from student-led or supplementary models. Evidence examining recently graduated doctors or alumni acting as near-peer educators within the formal, compulsory delivery of undergraduate curricula remains limited. In particular, little research has evaluated how embedding graduate near-peer tutors within structured, assessment-aligned core teaching influences learning experiences. An undergraduate Obstetrics and Gynecology (O&G) course at Mohammed Bin Rashid University of Medicine and Health Sciences in Dubai, United Arab Emirates, has previously been evaluated using cognitive load theory, focusing on curriculum design and simulation-based education rather than the instructional role of clinical tutors. That evaluation demonstrated favorable cognitive load outcomes within a stable course framework. Since then, the instructional role of clinical tutors has expanded within the same structure, creating an opportunity to examine whether formally embedded graduate near-peer involvement is associated with differences in perceived cognitive load and learning outcomes.

Aim

To compare student feedback and perceived cognitive load across two academic cycles of an undergraduate O&G course and to examine whether observed differences were associated with an expansion in the formal instructional role of clinical tutors.

Methods

This retrospective comparative educational evaluation analyzed routinely collected, anonymized student feedback from scheduled teaching sessions delivered between October and January across two academic cycles. Perceived cognitive load was assessed using a standardized questionnaire measuring intrinsic cognitive load, extraneous cognitive load, and germane cognitive load or self-perceived learning. Descriptive statistics and between-cycle comparative analyses were performed using a t-test. Free-text comments were reviewed descriptively to contextualize quantitative findings, with particular attention to tutor-related and facilitation-related themes.

Results

A total of 102 feedback responses from Cycle A and 130 responses from Cycle B were included. Compared with Cycle A, Cycle B demonstrated higher intrinsic cognitive load and higher germane cognitive load or self-perceived learning scores, while extraneous cognitive load remained consistently low across both cycles. Session-level analyses showed similar directional patterns, with statistical significance varying by session, reflecting differences in sample size and session coverage. Descriptive review of free-text comments indicated increased recognition of clinical tutors’ instructional visibility and facilitative role in the later cycle.

Conclusion

Differences in perceived intrinsic and germane cognitive load were observed between academic cycles within a largely stable course structure. These findings are associated with an expanded instructional role of embedded graduate clinical tutors but do not establish causality. The results highlight a gap in the literature regarding formally integrated graduate near-peer teaching models and support further focused investigation of their role within undergraduate medical curricula.

## Introduction

Medical education increasingly emphasizes the importance of structured learning environments that promote engagement, psychological safety, and effective knowledge acquisition. Within undergraduate medical curricula, formal teaching is traditionally delivered by senior faculty, while peer-assisted learning initiatives are commonly described as informal, voluntary, or extracurricular activities [1-3]. Peer-assisted learning is relevant to global health because it provides a cost-effective, scalable approach to medical education that helps address faculty shortages, expand training capacity, and build sustainable healthcare workforces in resource-limited settings. 

Although peer and near-peer teaching models are well established in the literature, their implementation and evaluation within compulsory, curriculum-embedded teaching remain limited [3,4]. More specifically, the literature around clinical tutors (recently graduated doctors who are alumni of the same medical program and who function as near-peer teachers within the formal undergraduate curriculum) is even more limited. These clinical tutors embedded within scheduled, compulsory teaching sessions are part of the official teaching team and contribute directly to course delivery alongside faculty. This role is distinct from informal “buddy” systems or extracurricular peer-assisted learning programs, as clinical tutors in this context are integrated into the official structure, objectives, and assessment-aligned teaching of the course. 

Peer-assisted and near-peer learning in medical education has demonstrated consistent benefits, including improved learner comfort, engagement, and perceived approachability of instructors [1,5]. However, most published studies focus on senior medical students teaching junior students, frequently through voluntary or supplementary initiatives [3,4,6]. Comparatively few studies examine recently graduated doctors acting as near-peer tutors, and even fewer evaluate such roles when they are embedded within the core delivery of compulsory undergraduate courses [2,7]. As a result, there is a clear gap in the literature regarding the educational impact of formally integrated, graduate-level near-peer teaching roles. Graduate-level near-peers bring the perspective of teaching after having experienced clinical practice. Addressing this gap is important as using recently graduated doctors in formal teaching roles offers scalable solutions for medical education at a time when the world faces a projected 10-11 million health-worker shortage by 2030 [8], making efficient, workforce-expanding training models critical to improving global health outcomes and access to care. 

Embedded clinical tutors may influence students’ learning experiences for several theoretical reasons. Near-peer instructors are often perceived as more approachable, which can enhance psychological safety and encourage engagement [1,5]. Clinical tutors occupy a unique position: they combine the clinical perspective of qualified doctors with recent experience of the same curriculum. This dual perspective may help them anticipate common difficulties, identify high-load concepts, and deliver explanations that are appropriately paced and tailored to learners’ needs. Such factors may reduce anxiety and support more effective learning, reflected in lower extraneous cognitive load (EL), higher germane load (GL) or self-perceived learning, and improved perceptions of session usefulness [9,10]. 

Cognitive load theory provides a useful framework for evaluating these environments. It describes the mental effort placed on working memory and distinguishes between intrinsic load (IL; task complexity), EL (instructional design), and GL (meaningful learning) [11,12]. In medical education research, these measures are used to assess instructional clarity, learning support, and perceived effectiveness. In this study, cognitive load theory is applied as an evaluative lens rather than as an intervention. 

An undergraduate Obstetrics and Gynecology (O&G) course at Mohammed Bin Rashid University has previously been evaluated using cognitive load theory, showing favorable outcomes related to program design and simulation-based teaching [7]. However, the role of clinical tutors was not a primary focus. Since then, the course structure has remained largely unchanged, while tutors have taken a more active role in teaching delivery, including overview sessions and formative activities such as multiple-choice question (MCQ) reviews that were previously faculty-led. This shift within a stable course framework created an opportunity to compare student-reported outcomes across two academic cycles using the same evaluation instrument. 

Accordingly, the aim of this study was to conduct a retrospective, comparative evaluation of an undergraduate O&G course across two academic cycles to examine whether changes in the formal role of clinical tutors were associated with differences in students’ perceived cognitive load and self-reported learning outcomes, as measured through routine student feedback data. 

## Materials and methods

Ethics 

This study used retrospective, anonymized educational feedback data collected as part of routine institutional evaluation. There was no direct participant contact and no identifiable data collected. Formal ethical approval was therefore not required. 

Study design and setting 

This study was a retrospective cross-sectional evaluation of anonymized student feedback collected during an undergraduate O&G clerkship across two academic cycles. The evaluation compared perceived cognitive load and self-perceived learning outcomes between a baseline cycle (Cycle A) and a subsequent cycle following an expansion in the instructional role of clinical tutors (Cycle B). 

The study was conducted at Mohammed Bin Rashid University of Medicine and Health Sciences (MBRU), Dubai, United Arab Emirates, within the undergraduate O&G clerkship of the MBBS program. The overall structure of the clerkship, including centralized teaching sessions, simulation activities, and formative assessments, remained largely consistent across academic cycles and has been previously described in detail in a study evaluating this course focusing on the impact of simulation-based teaching on cognitive load [7]. Since the study examines the same course, many elements of the methods, such as the course description, remain unchanged. To avoid duplication, only elements relevant to the comparative evaluation were described in this study. 

Study period and rotation structure 

Both academic cycles covered the same calendar time window, from October to January. This period was selected to correspond with the introduction and early implementation of the expanded clinical tutor role in Cycle B (2025/26), and the identical time window was examined in Cycle A (2024/25) to ensure temporal comparability. During each academic cycle, the study period spanned three consecutive clerkship rotations, rather than two complete rotations. Due to the alignment of rotation schedules with the selected time window, not all teaching sessions within each rotation fell within the study period. Specifically, for both Cycle A and Cycle B: 

Rotation 1 contributed feedback data from the revision session only. 

Rotation 2 contributed feedback data from all eight scheduled teaching sessions. 

Rotation 3 contributed feedback data from the induction session only. 

As a result, most teaching sessions contributed a single set of feedback responses per academic cycle, while induction and revision sessions contributed two sets of feedback responses per cycle. This structure ensured that identical session types and time points were included for both cycles, which is illustrated in Table 1. 

**Table 1:** Distribution of teaching sessions included in the analysis across rotations and academic cycles x = sessions included in the analysis.

	Week 1	Week 2	Week 3	Week 4	Week 5	Week 6	Week 7	Week 8
	Introduction	Obstetric Emergencies	Gynecology Emergencies	Labor and Delivery	Infertility	Medical condition in Pregnancy	Gynecology Oncology	Revision
Rotation 1								x
Rotation 2	x	x	x	x	x	x	x	x
Rotation 3	x							
Total sessions analyzed	2	1	1	1	1	1	1	2

Participants 

Different student cohorts participated in each rotation. As feedback was collected anonymously and could not be linked to individual students, it was not possible to determine whether individual students submitted feedback across multiple sessions. Baseline academic metrics (e.g., grade point average (GPA)), demographic variables, and prior cognitive load measures were not available for comparison due to anonymization of the dataset. Because responses could not be linked at the individual level, hierarchical or mixed-effects modeling to account for potential within-student clustering was not feasible. All responses were therefore treated as independent observations. Participant numbers and rotation sizes are summarized in Table 2.

**Table 2:** Number of students per rotation for each cycle

	Cycle A	Cycle B
Rotation 1	12	16
Rotation 2	11	14
Rotation 3	12	16

Inclusion and exclusion criteria 

All anonymized student feedback questionnaire responses submitted following scheduled teaching sessions during the study periods were included. There were no exclusion criteria. The feedback questionnaire required completion of all items prior to submission; therefore, no partially completed questionnaires were included, and there were no missing item-level data. 

Exposure of interest: Clinical tutor role 

The exposure of interest was the documented change in the instructional role of clinical tutors between the two academic cycles. 

In Cycle A, clinical tutors were alumni doctors from the 2021 and 2022 graduating cohorts. Their responsibilities primarily included administrative coordination, facilitation support during teaching sessions, and assistance during tutorials and simulation activities. Although tutors were present during teaching sessions, their involvement in direct instructional delivery was limited. Overview lectures were brief, and MCQ discussions were primarily led by consultant faculty. 

In Cycle B, the role of clinical tutors was expanded and more actively embedded within formal teaching delivery. Tutors were also recent alumni and assumed increased instructional responsibilities, including delivery of longer and more detailed overview lectures and active facilitation of MCQ discussion sessions. It is noteworthy that the clinical tutors in Cycle A and Cycle B were different individuals from different graduating cohorts. While both groups comprised recent alumni doctors of the same institution, the tutors serving in Cycle B were not the same individuals as those in Cycle A. The overall course structure, scheduling, number of sessions, and simulation format remained unchanged between cycles. 

The expansion of the clinical tutor role was defined descriptively based on course documentation, faculty communications, teaching materials, and tutor role descriptions, rather than through predefined quantitative fidelity metrics. Although tutors in Cycle B delivered longer overview segments and assumed increased responsibility in MCQ facilitation, the proportion of session time delivered by tutors was not formally timed or quantified, and no structured rubric or objective measurement of tutor involvement was applied.

Session-level analyses, therefore, focused on sessions in which tutor instructional involvement was most substantively expanded according to course documentation. The exposure variable reflects a naturalistic curricular evolution within routine educational practice rather than a standardized intervention.

Data source and questionnaire 

Data were obtained from a standardized, anonymous online student feedback questionnaire routinely administered by the university following each teaching session. The questionnaire comprised both quantitative and qualitative components. The quantitative domain used an expanded cognitive load scale developed by Leppink et al. [9]. This is a widely validated self-report tool measuring IL, EL, and GL. Students were informed that the questionnaire assessed their cognitive load during the session and were asked to rate each item on a scale from 0 (not at all the case) to 10 (completely the case). The scale measured IL, EL, and GL, or self-perceived learning. The qualitative domain invited free-text comments. The questionnaire structure is summarized in Table 3. 

**Table 3:** Questionnaire used CL, cognitive load; IL, intrinsic load; EL Ins, instructional extraneous load; EL Noi, environmental extraneous load; EL Dev, device-related extraneous load; GL/SPL, Germane load/self-perceived learning.

	Student Feedback Questionnaire Used	CL Component Assessed
1	The topics covered in the session were very complex	IL
2	The session covered theories that I perceived as very complex	IL
3	The session covered concepts and definitions that I perceived as very complex	IL
4	The instructions and/or explanations during the session were very unclear	EL Ins
5	The instructions and/or explanations during the session were in terms of learning very ineffective	EL Ins
6	The instructions and/or explanations during the session were full of unclear language	EL Ins
7	Low-quality audio made the instructions hard to follow	EL Ins
8	Noises in the environment made it difficult to focus on the learning content	EL Noi
9	Distractions in the environment made learning ineffective	EL Noi
10	Unrelated events occurring in the environment made it difficult to focus	EL Noi
11	My activities on my phone/computer made it difficult to focus on the learning content	EL Dev
12	Messages and notifications from my phone/computer made learning unclear	EL Dev
13	Others’ phone/computer use distracted me, making it hard to learn	EL Dev
14	Technical issues made learning ineffective	EL Dev
15	Problems with technology made it difficult to focus	EL Dev
16	The session really enhanced my understanding of the topic(s) covered	GL/SPL
17	The session really enhanced my knowledge and understanding of obstetrics and gynecology	GL/SPL
18	The session really enhanced my understanding of the theories covered	GL/SPL
19	The session really enhanced my understanding of concepts and definitions	GL/SPL
20	Please feel free to include any free text comments below	

All items were scored on a numerical scale from 0 to 10. Feedback was collected immediately following each teaching session.

Outcomes 

The primary outcomes were mean and median scores for IL, EL, and GL domains at the overall cycle level (A vs B). Secondary outcomes included session-level comparisons of cognitive load domains and descriptive awareness of free-text comments. In addition to overall cycle-level comparisons, selected session-level analyses were performed. These focused on sessions known to be associated with higher IL demands and sessions in which the expansion of the clinical tutor role was most pronounced. 

Statistical analysis 

Statistical analysis was performed using IBM SPSS 29.0 Statistical software (IBM Corp., Armonk, NY). Cognitive load items were grouped by domain, and descriptive statistics, including mean, standard deviation, and median, were calculated at both the session level and across all sessions within each academic cycle. Comparative analyses between Cycle A and Cycle B were conducted using independent-samples t-tests. Statistical significance was defined as a two-sided p-value of less than 0.05. As feedback data were anonymized and individual identifiers were unavailable, responses were treated as independent observations. 

Qualitative data handling 

Free-text comments submitted as part of the standard post-session feedback questionnaire were analyzed descriptively by artificial intelligence (AI) to provide contextual insight into students’ perceptions of the teaching sessions. This analysis was intended to complement the quantitative cognitive load findings and was not designed to constitute a formal qualitative thematic analysis. 

Comments from each academic cycle were analyzed separately using a natural language processing tool (ChatGPT, OpenAI, San Francisco, CA) to generate high-level summaries of commonly expressed perceptions. No predefined coding framework was applied, and no assessment of inter-rater reliability was performed. 

The qualitative summaries were reviewed by the authors to ensure consistency with the original feedback and to verify that no identifiable information was retained. The qualitative findings were used solely to contextualize the quantitative results, particularly in relation to changes in the visibility and instructional role of clinical tutors across academic cycles. No causal inferences were drawn from the qualitative data. 

## Results

Dataset overview 

A total of 102 anonymized student feedback responses were included in Cycle A and 130 responses in Cycle B. Responses were collected during the October-January study period across three clerkship rotations per academic cycle. Due to the structure of the study window, most teaching sessions contributed a single set of feedback responses per cycle, while induction and revision sessions contributed two sets. The distribution of sessions and responses included in the analysis is summarized in Table 4.

**Table 4:** Comparison of cognitive load domains between academic cycles

Cognitive Load Domain	Cycle A Mean (SD)	Cycle B Mean (SD)	p-Value
Intrinsic load (mean)	1.32 (0.88)	3.22 (2.62)	0.001
Intrinsic load (median)	1.26 (0.78)	3.32 (2.91)	0.001
Extraneous load -- Instruction (mean)	1.28 (0.73)	1.25 (0.96)	0.369
Extraneous load -- Instruction (median)	1.21 (0.68)	1.19 (0.94)	0.434
Extraneous load -- Noise (mean)	1.17 (0.55)	1.26 (1.03)	0.223
Extraneous load -- Noise (median)	1.14 (0.53)	1.28 (1.13)	0.112
Extraneous load -- Devices (mean)	1.13 (0.51)	1.13 (0.82)	0.497
Extraneous load -- Devices (median)	1.09 (0.47)	1.12 (0.82)	0.383
Germane load/SPL (mean)	9.09 (1.55)	9.58 (1.07)	0.003
Germane load/SPL (median)	9.24 (1.52)	9.62 (1.05)	0.012

All questionnaire items were fully completed in all submitted responses, and there were no missing item-level data. As responses were anonymized, individual students may have contributed feedback across multiple sessions; responses were therefore treated as independent observations. 

Overall cognitive load comparisons 

At the overall cycle level, statistically significant differences were observed in IL and GL between academic cycles, while EL remained low and unchanged. IL was significantly higher in Cycle B compared with Cycle A. GL and self-perceived learning were also significantly higher in Cycle B. In contrast, instruction-related, noise-related, and device-related EL domains showed no statistically significant differences between cycles. Overall domain-level comparisons are presented in Table 4. 

Session-level analyses

Session-level analyses were performed for four of the eight scheduled teaching sessions. These sessions were selected because they represented the sessions in which the expansion of the clinical tutor instructional role was most substantial, including increased involvement in overview teaching and discussion-based components. The remaining sessions were primarily unchanged in structure and tutor involvement and were therefore not included in a detailed session-by-session comparative analysis. Across several of these sessions, IL scores were higher in Cycle B, with statistically significant differences observed in selected sessions, including induction, labor and delivery, obstetric emergencies, and revision sessions. GL scores were consistently higher in Cycle B across analyzed sessions; however, not all session-level comparisons reached statistical significance. This was particularly evident in sessions for which only a single set of feedback responses was available per cycle, limiting statistical power. 

Across all analyzed sessions, EL remained low and comparable between academic cycles. Session-level comparisons are summarized in Table 5.

**Table 5:** Session-level comparison of selected teaching sessions

Session	Cognitive Load Domain	Cycle A Mean (SD)	Cycle B Mean (SD)	p-Value
Induction session	Intrinsic load	1.11 (0.62)	2.34 (1.91)	0.041
	Germane load/SPL	8.96 (1.48)	9.51 (1.02)	0.118
Labor & delivery	Intrinsic load	1.33 (0.88)	3.71 (2.73)	0.027
	Germane load/SPL	8.83 (1.88)	9.64 (0.75)	0.304
Obstetric emergencies	Intrinsic load	1.50 (0.77)	4.31 (2.80)	0.004
	Germane load/SPL	8.90 (1.64)	9.64 (0.71)	0.057
Revision session	Intrinsic load	1.23 (0.87)	2.86 (2.65)	0.008
	Germane load/SPL	9.16 (1.55)	9.66 (1.00)	0.164

Free-text feedback analysis 

Free-text comments from both academic cycles were analyzed using AI-assisted natural language summarization to provide a descriptive thematic overview. 

Cycle A (baseline) comments reflected highly positive perceptions of the sessions, which were frequently described as well-organized, informative, and confidence-boosting. Students particularly valued revision sessions prior to clinical placements, objective structured clinical examination (OSCE) and short-answer question (SAQ) practice, and practical feedback during structured activities. Feedback emphasized preparedness for examinations and clinical rotations. Mentions of clinical tutors in Cycle A primarily related to their roles in practical support, OSCE facilitation, and feedback during skills-based components. Suggested areas for improvement were largely logistical, including room size, seating arrangements, and the pacing of concept-heavy obstetrics content. 

Cycle B (post-expansion) comments were overwhelmingly positive and consistently described the rotation as one of the most organized and effective learning experiences students had encountered. Students highlighted the strong integration of overview lectures, MCQ discussions, and OSCE practice, noting that this structure enhanced understanding and exam preparation. Teaching sessions were frequently described as interactive, supportive, and non-judgmental. Mentions of clinical tutors in Cycle B extended beyond practical facilitation to include active teaching roles within overview lectures and MCQ sessions. Students frequently referenced clarity of explanations, approachability, and structured guidance as key strengths. Areas for improvement again focused on logistics and delivery optimization, such as sharing materials in advance, time management, room layout, and increasing opportunities for hands-on practice. 

Across both cycles, qualitative feedback aligned with quantitative findings, with consistently low references to confusion, environmental distractions, or instructional disorganization, with comments reflecting greater instructional visibility of tutors. 

## Discussion

Peer-assisted learning is highly relevant to global health because it offers a cost-effective, scalable approach to medical education in the face of faculty shortages and limited training capacity. Expanding formal teaching roles to recently graduated doctors can increase educational capacity while strengthening the workforce -- an urgent priority as the world faces a projected shortfall of 10-11 million health workers by 2030. This retrospective comparative evaluation examined differences in perceived cognitive load and learning outcomes across two academic cycles of an undergraduate O&G clerkship following an expansion in the instructional role of clinical tutors. Overall, differences were observed in IL and GL between cycles, while EL remained consistently low. These findings suggest changes in how students experienced the instructional environment over time, although causal attribution cannot be established. 

Contrary to the initial hypothesis that IL would decrease with increased instructional support, IL scores were higher in the later academic cycle. While increased IL is sometimes interpreted as undesirable, cognitive load theory conceptualizes IL as reflecting the inherent complexity and element interactivity of learning material rather than instructional [10,13]. Very low IL may occur when tasks are overly simple, insufficiently challenging, or highly familiar to learners, which may reduce engagement and lead to superficial processing or boredom rather than meaningful learning [14]. From this perspective, the observed increase in IL may reflect greater exposure to complex or information-dense content as expected when instructional materials are reviewed and upgraded annually.

Importantly, the increase in IL occurred alongside higher GL and self-perceived learning scores. GL represents productive cognitive effort directed toward schema construction and integration into long-term memory [9]. When increases in IL are accompanied by increases in GL, while EL remains low, the pattern is consistent with deeper cognitive engagement and more effective learning rather than reduced instructional quality [10,12]. Educational theory emphasizes that IL should be optimized rather than minimized to zero, allowing learners to allocate cognitive resources toward meaningful learning processes rather than disengagement [10,12]. Furthermore, higher GL and self-perceived learning have been associated with improved academic performance, supporting the interpretation that this pattern may represent an educational improvement rather than overload [15].

EL related to instructional clarity, environmental distractions, and technology remained low and stable across both academic cycles. This stability suggests that differences in IL and GL were unlikely to be driven by increased confusion, environmental noise, or instructional disorganization. Instead, the findings may reflect changes in instructional depth or learner engagement within a similarly structured learning environment. 

One plausible explanation for these findings relates to the expanded role of clinical tutors embedded within formal, compulsory teaching sessions. Clinical tutors in this program are recent graduates and alumni who are fully qualified doctors, while also having recent lived experience of the same curriculum. Their increased involvement in overview lectures and MCQ facilitation may have contributed to improved pacing, targeted clarification of high-load concepts, and enhanced learner support. Near-peer educational models have previously been associated with increased approachability and psychological safety, which may facilitate deeper engagement with complex material and greater investment in learning-relevant cognitive processes [2]. 

Notably, unlike most near-peer or peer-assisted learning literature, which focuses on senior medical students teaching juniors in informal or extracurricular contexts, the clinical tutors in this study were embedded within the core curriculum and formed part of the official teaching team. This distinction is important, as it represents a formalized graduate near-peer role within scheduled, compulsory sessions. The present study, therefore, contributes preliminary evidence addressing a gap in the literature regarding formally embedded graduate near-peer tutors, particularly within undergraduate medical education in the Middle East, where such models remain under-represented. 

Several limitations must be acknowledged. This was a retrospective, non-randomized evaluation comparing two different student cohorts, which limits causal inference. Baseline academic performance, demographic variables, and prior cognitive load measures were not available due to anonymization of the dataset, and therefore, no adjustment for baseline equivalence between cohorts was possible.

Feedback responses were collected anonymously at the session level and could not be linked to individual students. As a result, repeated measures from the same student across multiple sessions could not be accounted for using hierarchical or mixed-effects modeling. Responses were treated as independent observations, which may underestimate variance and potentially inflate statistical significance. Findings should therefore be interpreted conservatively and as associative rather than causal.

The qualitative component was descriptive and exploratory in nature. No formal coding framework, audit trail, reflexivity statement, triangulation strategy, or inter-rater reliability assessment was applied. Qualitative findings were used solely to contextualize quantitative results and should not be interpreted as the product of formal thematic analysis.

Finally, the expansion of the clinical tutor role was operationalized descriptively rather than quantified using objective fidelity measures (e.g., percentage of session time delivered by tutors or number of MCQs facilitated). While course documentation confirmed increased instructional involvement, the absence of standardized exposure measurement limits precision in attributing observed differences specifically to tutor role expansion.

Despite these limitations, the findings provide a rationale for further investigation. Future studies could adopt prospective or mixed-methods designs, incorporate tutor-specific evaluation items, and explore relationships between cognitive load measures and objective learning outcomes. Qualitative interviews with students and tutors may further clarify how formally embedded graduate near-peer roles influence learning experiences and instructional effectiveness. 

## Conclusions

This retrospective comparative evaluation found that expanding the instructional role of recent-graduate clinical tutors within compulsory undergraduate O&G teaching was associated with higher IL and GL, while EL remained consistently low. Together, this pattern may reflect greater cognitive engagement and enhanced self-perceived learning within an otherwise stable course structure. However, because the study was retrospective and non-randomized, these findings indicate association rather than causation.

The findings address a substantial gap in the medical education literature regarding formally embedded graduate near-peer teaching roles. Unlike most peer-assisted learning models, which are informal, voluntary, or student-led, the clinical tutors evaluated in this study were qualified doctors integrated into the official teaching team and curriculum delivery. Taken together, these findings support the need for more targeted, prospective investigation into the educational impact of formally embedded graduate near-peer tutors. If further research confirms a positive effect on learning outcomes, this model may represent a scalable and sustainable approach to strengthening undergraduate medical education through optimized use of recent-graduate teaching capacity. Research on the use of clinical tutors and near-peer teachers is essential, as it can inform scalable, cost-effective training models that expand educational capacity, strengthen the health workforce, and help address pressing global health priorities. Evidence from other settings suggests that engaging recent graduates as peer educators may enhance learning experiences while also representing an efficient use of educational resources, particularly in contexts with limited faculty teaching capacity. Further investigation of formally embedded graduate near-peer teaching is warranted, as this model may offer a pragmatic approach to enhancing educational delivery while optimizing existing human resources in an increasingly constrained global medical education landscape.
